# Supplementary material for: Photodynamic Therapy in Combination with the Hepatitis B Core Virus-like Particles (HBc VLPs) to Prime Anticancer Immunity for Colorectal Cancer Treatment
Source: Cancers (Basel). 2022 May 31;14(11):2724. doi: 10.3390/cancers14112724 (PMC9179923; doi:10.3390/cancers14112724)
Supplement: Supplementary file 1 [file cancers-14-02724-s001.zip › cancers-1721775-supplementary.pdf]

Photodynamic Therapy in Combination with the Hepatitis B Core Virus-like Particles (HBc VLPs) to Prime Anticancer Immunity for Colorectal Cancer Treatment (Supporting Information)

Yang Hao [1], Zili Gu [1], Zhenfeng Yu [1], Timo Schomann [1,2], Sana Sayedipour [1], Julio C. Aguilar [3], Peter ten Dijke [4,*] and Luis J. Cruz [1,*]

[1] Translational Nanobiomaterials and Imaging (TNI) Group, Department of Radiology, Leiden University Medical Center, Albinusdreef 2, 2333 ZA Leiden, The Netherlands; y.hao@lumc.nl (Y.H.);
z.gu@lumc.nl (Z.G.); z.yu@lumc.nl (Z.Y.); t.schomann@lumc.nl (T.S.);
s.s.sayedipour@lumc.nl (S.S.)

[2] Percuros B.V., Zernikedreef 8, 2333 CL Leiden, The Netherlands

[3] Center for Genetic Engineering and Biotechnology, Center for Genetic Engineering and Biotechnology, Havana 10600, Cuba; julio.aguilar@cigb.edu.cu

[4] Department of Cell and Chemical Biology and Oncode Institute, Leiden University Medical Center,
Einthovenweg 20, 2300 RC Leiden, The Netherlands

* Correspondence: p.ten_dijke@lumc.nl (P.t.D.); l.j.cruz_ricondo@lumc.nl (L.J.C.); Tel.: +31-71-526-9271 (P.t.D.); +31-71-5265764 (L.J.C.)

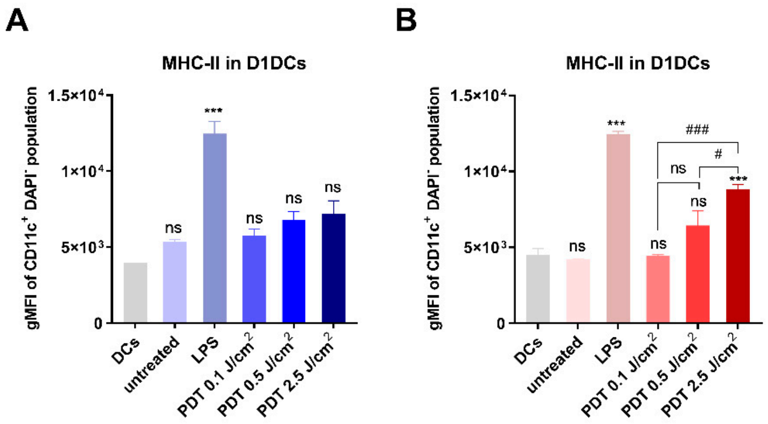

Figure S1 Untreated cancer cells and (A) MC-38 or (B) CT-26 treated with different doses of PDT were co-cultured with DCs for 24 h immediately post-treatment. The gMFI of MHC-II in DCs ($CD11c^{+}DAPI^{-}$ cells) were compared to untreated DCs control group (the statistical differences are denoted as *$p < 0.05$, *** $p < 0.001$, # $p < 0.05$, ### $p < 0.001$, ns: non-significantly).

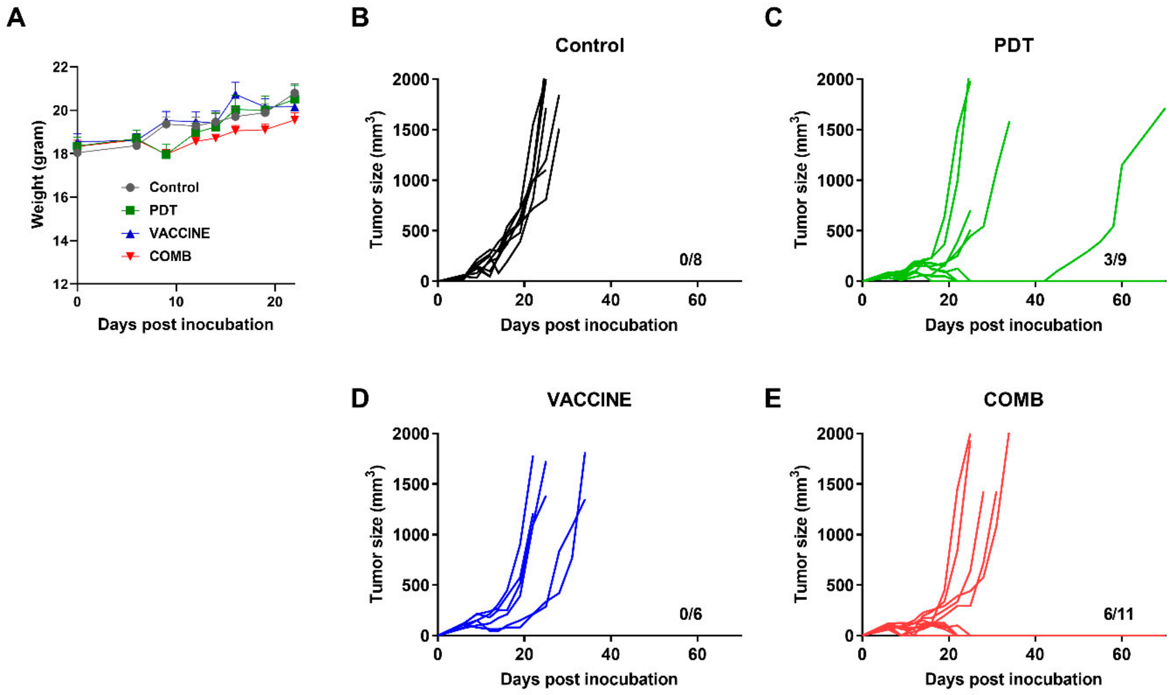

Figure S2 Effects of FOSCAN-PDT combined with HBc VLPs *in vivo*. (A) Weight changes and individual tumor growth curve (B, C, D, and E) of MC-38 tumor-bearing mice, which received different treatments: either PBS (mock control), FOSCAN-PDT, vaccine, or combined treatment (COMB), respectively.

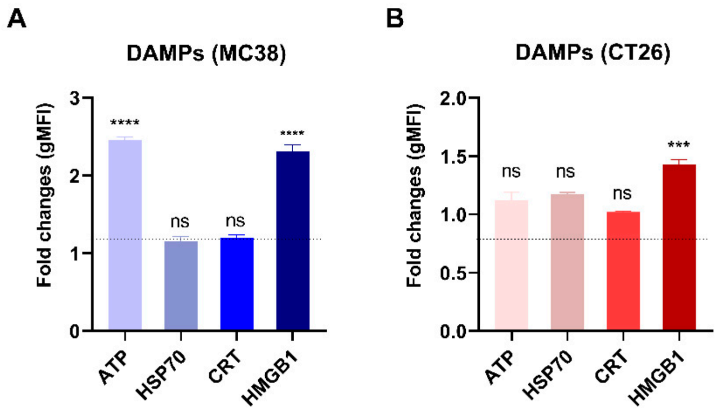

Figure S3 Crucial ICD marker change after FOSCAN-PDT treatment. (A) Fold changes quantification of flow cytometry analysis of CRT and HSP70 exposure on the surface of PDT-treated MC-38 tumor cells and ATP release. (B) Fold increase quantification of flow cytometry analysis of CRT and HSP70 exposure on the surface of PDT-treated MC-38 tumor cells and ATP release. All data shows the mean values ± SEM from three independent experiments. Statistical significance was calculated using the Student's *t*-test, by comparing experimental groups to the control (the statistical differences are denoted as *** $p < 0.001$ and **** $p < 0.0001$).

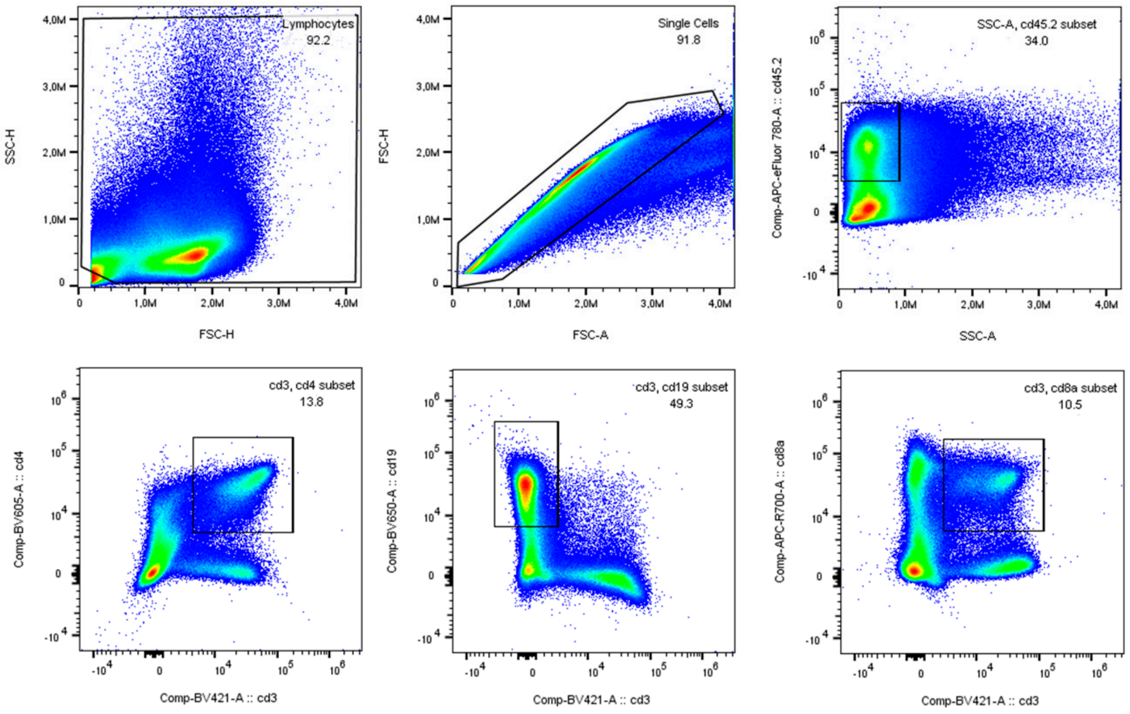

Figure S4 Flow cytometry gating strategy for splenocytes.
